# Prevalence and distribution of human papillomavirus genotypes in women with abnormal cervical cytology in Ethiopia: a systematic review and meta-analysis

**DOI:** 10.3389/fonc.2024.1384994

**Published:** 2024-10-15

**Authors:** Solomon Demis Kebede, Shegaw Zeleke, Amare Kassaw, Tigabu Munye Aytenew, Demewoz Kefale, Worku Necho Asferie

**Affiliations:** ^1^ Department of Maternity and Neonatal Nursing, College of Health Sciences, Debre Tabor University, Debre Tabor, Ethiopia; ^2^ Department of Nursing, College of Health Sciences, Debre Tabor University, Debre Tabor, Ethiopia; ^3^ Department of Pediatrics and Child Health Nursing, College of Health Sciences, Debre Tabor University, Debre Tabor, Ethiopia

**Keywords:** HPV genotypes, cervical lesion, abnormal cervical cytology, pap smear test, cervical cancer screening

## Abstract

**Background:**

Cervical cancer is the 4^th^ most common cancer in women globally. Determining the prevalence of the high-risk human papillomavirus (HR-HPV) and low-risk (LR-HPV) genotypes and the distribution in abnormal cervical cytology will be essential in a future population-based cervical cancer prevention program.

**Method:**

Primary studies with women with abnormal cervical cytology were systematically searched for in Medline, CINHAL, Google Scholar, African Journal Online, and the University of Antwerp repository from 19-30 May 2023. A weighted inverse-variance random effects model was used. Variations across the studies were checked using a forest plot, I^2^ statistics, and Egger’s test. Group analysis was performed for evidence of heterogeneity.

**Results:**

The pooled prevalence of human papillomavirus (HPV) genotypes with abnormal cervical cytology of a precancerous cervical lesion was 38.74% (95% CI: 27.56-49.93). The leading pooled prevalence estimates by subgroup analysis were 18% (95% CI: 13-26), 14% (95% CI: 111-16), and 66% (51-79) for women with retroviral infection (RVI), DNA genotyping with amplification, and central parts of Ethiopia respectively. There were 25 HPV variants identified by genotyping techniques with the five most prevalent HPV genotypes being HPV-16 and HPV-18 coexisting at 54%; HPV-16 alone at 29%; HPV-51 at 16%; HPV-52 at 13%; and HPV-31 and HPV-33 each contributing approximately 12%.

**Conclusion:**

The pooled prevalence of HPV genotypes was higher than in other countries. HPV-51, HPV-52, HPV-31, and HPV-33 are the most prevalent genotypes. Hence, the nonavalent vaccine type would be the one that includes all the most prevalent HPV genotypes, but HPV-51in Ethiopia. Additional data on similar DNA test techniques for comparisons with precancerous lesions and invasive cancer are needed. Cervical cancer prevention and control programs in Ethiopia should be aligned with the most prevalent genotypes.

**Systematic review registration:**

https://www.crd.york.ac.uk/prospero/, identifier CRD42023428955.

## Background

Cervical cancer is the most prevalent type of cancer affecting the reproductive organs of women and the primary cause of cancer-related deaths in low- and middle-income countries (LMICs), such as Ethiopia. For instance, the ten African countries with the highest rates of cervical cancer were all above the global average, at 13.30 per 100,000 women and 604,127 cases (1–3).

Africa has the highest incidence of cervical cancer worldwide with rates of 31.6 cases per 100,000 people which is above the global incidence of 13.3 cases per 100,000 people (2, 4).

A systematic population-based program with pap tests has been reducing the incidence of invasive cervical cancer in high-income countries by detecting and treating cervical lesions. However, screening is limited in low-income countries as it is being performed in public or private laboratories in urban areas for only approximately 5% of eligible women. In addition, the absence of a well-organized surveillance and review system results in poor screening or lack of follow-up (2, 5–7).

Evidence suggests that less than 5% of all eligible women in developing countries receive cytology-based screening within 5 years. This is because there are few healthcare providers and professionals involved in such analyses or because of the limited availability of medical facilities available to accommodate the demand for screening and treatment. Moreover, in LMICs, cytology services are limited to teaching hospitals or private clinics in larger cities and are not accessible to all eligible women (5, 6).

The Bethesda system is a standardized model for reporting cervicovaginal cytology by which there are low-grade squamous intraepithelial lesions (LSILs), high-grade squamous intraepithelial lesions (HSILs), or atypical squamous cells [of undermined significance (ASCUS) or cannot rule out HSIL (ASC-H)] (8–10). Approximately 15% of human papillomavirus (HPV) infections progress to low LSILs within 3-4 years, and 30-70% of LSILs advance to HSILs in 10 years (11–13). The most common HPV types identified in previous studies were 16, 18, 31, 33, 35, 39, 45, 51, 52, 56, 58, 68, and 59, which were considered high risk. Several groups, including HPV types 53, 66, 70, 73, and 82, have been classified as potential or high-risk types. Approximately seven types of high-risk human papillomavirus (HR-HPV) are associated with approximately 87% of cervical cancer cases worldwide. Forty types of papillomaviruses, which tend to spread to the genitals, usually infect the cervix, genitals, urethra, and anus in both sexes (1, 7, 14).

According to the WHO global strategy to accelerate the elimination of cervical cancer as a public health problem by 2030, 90% of girls will be fully vaccinated against HPV by the age of 15 (5). There are currently two types of HPV vaccines licensed in many countries, and these vaccines have been proven to prevent more than 95% of HPV infections caused by HPV types 16 and 18, which cause 70% of cancer cases (15). However, there is no consistent information on which type of vaccine is better at preventing HPV-related cervical cancer in Ethiopia. This review aimed to determine the prevalence, most specific type, and distribution of HPV genotypes among women with abnormal cervical cytology.

## Materials and methods

### Reporting

The results of this review were reported in accordance with the MOOSE checklist for meta-analyses of observational studies (16). **Supplementary File 1** shows the MOOSE checklist.

The review has been registered with PROSPERO ID: CRD42023428955.

### Search strategy and source of information

Data searching was conducted from 19-30 May 2023. The articles retrieved were published from 2006 to 2023, were written in English, and had cross-sectional and cohort study designs. The MEDLINE, Web of Science, Scopus, Google Scholar, Africa Online Journals, University of Antwerp repository, and gray literature databases were searched. The key search terms and phrases used were “human papillomavirus”, “human papillomavirus DNA tests”, “human papillomavirus investigating”, “cervical cancer”, “precancerous cervical lesion”, “cervical tumor”, “cervical malignancy”, “reproductive women”, “adolescent girls”, “mothers”, and “Ethiopia”. The search strategy was developed using various Boolean operators. Hence, to fit the advanced PubMed database, the following search strategies were applied on 29 March 2023: [(human papillomavirus screening [MeSH Terms]) AND (human papillomavirus testing [MeSH Terms] AND (human papilloma investigating [MeSH Terms] AND (cervical neoplasms [MeSH Terms]) OR (cervical cancer [MeSH Terms]) OR (precancerous cervical lesion [MeSH Terms]) OR (cervical tumor [MeSH Terms]) AND (reproductive women [MeSH Terms]) OR (adolescent girls [MeSH Terms]) AND (Ethiopia).

### Study selection

The studies were imported into Mendeley Desktop using data management software to eliminate duplicate data. Two independent reviewers reviewed the title and abstract. Differences between reviewers were checked by article-based analysis. Abstract and full-text analyses were performed by two independent authors in three groups. All reviewers screened all studies with discussions on inconsistency amendments among the reviewers.

### Eligibility criteria

#### Inclusion criteria

The primary studies included were those that reported both high-risk HPV (HR-HPV) and low-risk HPV (LR-HPV) genotype prevalence and distribution in women with LSILs or HSILs in Ethiopia.

#### Exclusion criteria

Articles without full text available and qualitative studies were excluded.

### Outcome measurement

The overall HPV prevalence was defined as the number of women with positive HPV tests among all women with LSIL or HSIL cytology reports, expressed as a percentage.

Similarly, the prevalence of HPV type specificity was defined as the number of women with positive HPV type-specific tests among all women with LSIL or HSIL cytology reports, expressed as a percentage.

### Quality assessment

The JBI quality appraisal criteria were used (17). The tool has nine main features. The first feature is suitability for the sample frame. The second is using the convenient sampling technique. Third, the sample size should be large enough. Fourth is a description of the research object and environment. Fifth, the data analysis program was sufficient. The sixth is the validity of the situation analysis method. The seventh feature is being reliable for all participants. Eight is the necessity of statistical analysis. The final feature is being reasonable and cost-effective.

Studies were considered low risk when five or more were positive out of the nine criteria. Two independent authors evaluated the quality of the studies. Disputes are resolved with the intervention of a third-party moderator. **Supplementary File 2** shows the JBI quality assessment of the included studies.

### Data extraction

The adapted PICO format was used to explicitly review the pieces of literature and clear specifications for the inclusion and exclusion criteria. The adapted PICO comprises Population (P), Exposure (E), Outcome (O), and Context (Setting) as described below.

- Population: women with abnormal cervical cytology
- Exposure: human papillomavirus (HPV)
- Outcome: prevalence and distribution
- Context (Setting): Ethiopia

Both authors (SD and TM) extracted the data using a standard method. The author, year, study area, study design, setting, sample size, and HPV type on abnormal cervical cytology were extracted. This step was repeated every time a change was found in the extracted data. If inconsistencies between the extracted data persisted, a third reviewer (SZ) was included.

### Statistical analysis

Statistical pooling for the prevalence proportion of estimates was performed according to the random effects model using Statistical software for Data Science (STATA V17). The random effects model of analysis was used since the studies identified were observational and had both clinical and methodological variability. The heterogeneity of the studies was evaluated based on Cochrane’s Q and I2 tests as well as the Q/df (degree of freedom) ratio. Thus, Cochrane’s Q test (p = 0.1), Q/df = 1, and I2 = 50% were considered cutoff points for identifying heterogeneity and selecting an effective model for analysis.

Forest plots were generated to present the pooled prevalence of HPV genotypes in women with precancerous cervical lesions. In line with this, subgroup analyses were carried out to explain HPV DNA variant distributions in subgroups with the potential to account for the differences in the effect sizes of the HPV genotypes. Egger’s regression tests were performed to objectively test for the presence of a small study effect.

## Results

### Selection of studies for review

**Figure 1:**
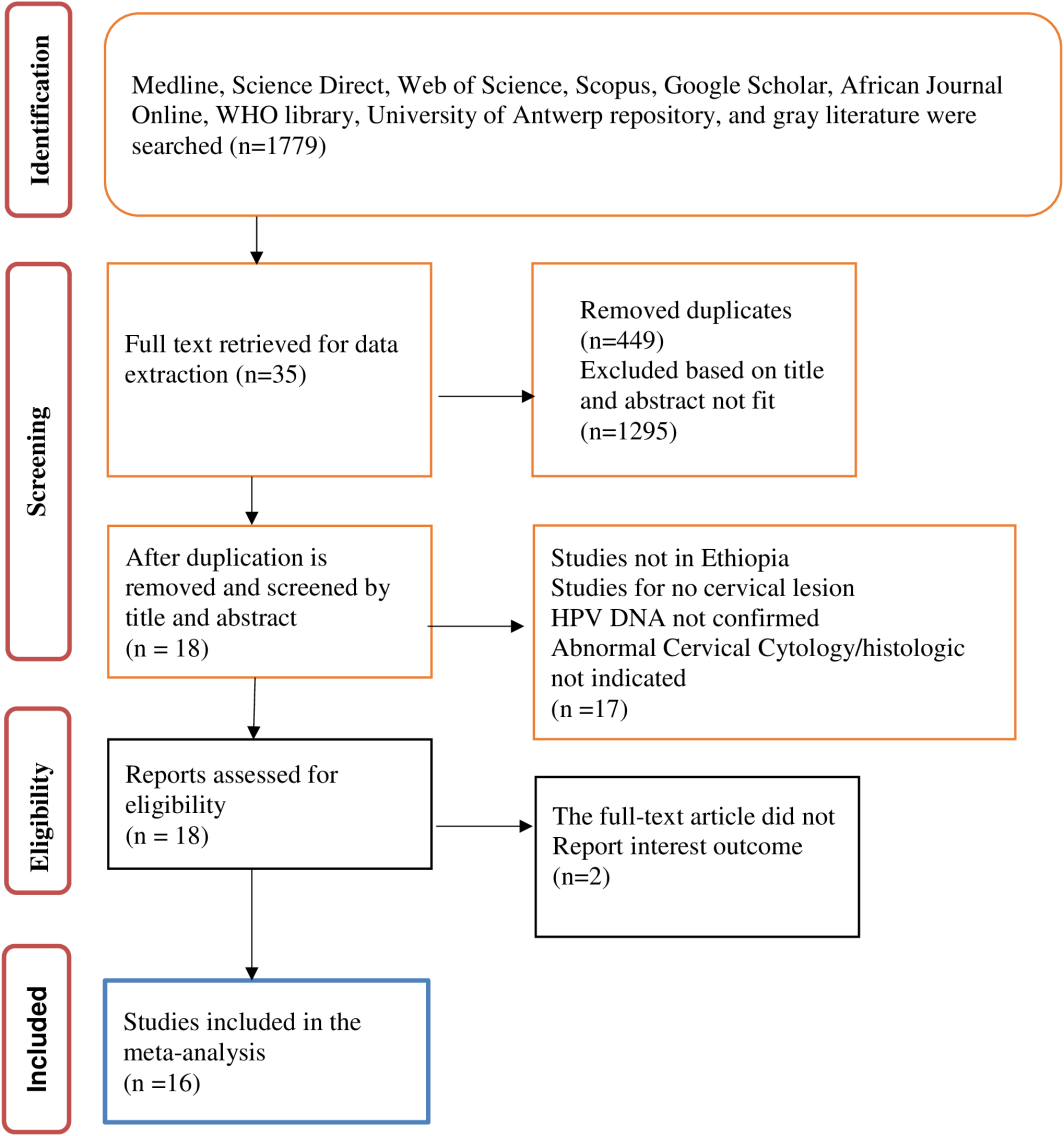
The study selection process.

A total of 1,779 research citations that met the requirements of the National Institute of Health (NIH) quality assessment tool for observational cohort and cross-sectional study guidelines were retrieved. Following the removal of duplicates and the screening of titles and abstracts, 35 studies were retrieved for full-text review. Of these, a further 17 were excluded as they were not full-length articles or did not report outcomes of interest. The remaining 18 full-text articles were assessed for eligibility and two were excluded as they did not report the outcome of interest. This left 16 studies included in the review and meta-analysis. Data were extracted by title before beginning the systematic screening using the PRISMA flow diagram for the final review of the included studies. **Figure 1** shows the PRISMA flow diagram of the study selection process.

### Characteristics of the included studies

For the systematic review, 16 studies were included from which three studies were from the Oromia region (18–20), three from Addis Ababa in central Ethiopia (21–23), two studies from northwest Ethiopia (15, 24), and one in each of Amhara region, Tigray, Southern nations nationalities, south-central Ethiopia, southwest Ethiopia, eastern Ethiopia, Armauer Hansen Research Institute, and gynecology referral hospitals in Ethiopia, respectively (4, 14, 15, 24–30). The age range of the women studied was 15-85 years (14, 19, 21, 22, 26, 27, 29, 30). The mean age of the women was 32 years in one study (18), and the mean age was 15- ≥ 44 years in another study (16).

A total of 5,276 study participants were included. Of these, 2,621 were infected with one or more HPV genotypes. **Table 1** shows the characteristics of the included studies.

**Table 1:** Characteristics of the included studies.

First author and year	Participants age range or mean	Health facility	Study area/region	Study design	Sample size	Population outcome	Prevalence	Quality status
Ali et al. (2019) (21)	18-64	Addis Ababa	Central Ethiopia	Cross-sectional	50	38	76.00	Low risk
Bartholomeusz and Locarnini (2006) (29)	21-85	Central	Armauer Hansen Research Institute,	Cross-sectional	149	136	91.28	Low risk
Bekele et al. (2010) (27)	32-65	Jimma	Southwest Ethiopia	Cross-sectional	83	68	81.93	Low risk
Bogale et al. (2022) (22)	25-49	Addis Ababa	Central Ethiopia	Cross-sectional	130	24	18.46	Low risk
Derbies et al. (2022) (31)	—	Bahir Dar	Amhara	Cohort	3633	1950	53.67	Low risk
Derbies et al. (2023) (14)	30-67	Gynecology referral clinics	Northwest Ethiopia	Cross-sectional	154	77	50.00	Low risk
Gebremariam (2016) (25)	—	Mekele	Tigray	Cohort	86	21	24.42	Low risk
Haile et al. (2019) (18)	32	Adama	Oromia	Cross-sectional	27	6	22.22	Low risk
Kiros et al. (2021) (24)	……	Debre Tabor Comprehensive Hospital	Northwest	Cross-sectional	109	14	12.84	Low risk
Lemma et al. (2022) (19)	30-35	Adama	Oromia	Cross-sectional	66	6	9.09	Low risk
Leyh-Bannurah et al. (2014) (4)	15-64	gurage zone	southern nations	Cross-sectional	86	21	24.42	Low risk
Megersa et al. (2023) (20)	15-≥44	hashemite	Oromia	Cross-sectional	143	21	14.69	Low risk
Mekuria et al. (2020) (23)	18-70	Addis Ababa	Central Ethiopia	Cross-sectional	164	28	17.07	Low risk
Seyoum et al. (2023) (28)	30-60	Harara, Dire Dawa and Jigjiga	Eastern Ethiopia	Cross-sectional	152	35	23.03	Low risk
Teka et al. (2021) (26)	30-49	Butajira rural	South-central Ethiopia	Cross-sectional	205	117	57.07	Low risk
Wolday et al. (2018) (30)	40.1-43.2	Gynecology referral clinics	Ethiopia	Cross-sectional	134	59	44.03	Low risk
	Total				5276	2621		

Regarding the type of HPV genotyping tests and techniques, two studies used DNA testing with direct genomic detection with hybrid capture (4, 24), eight used DNA testing with amplification for HR genotyping (14, 21, 22, 25–31), and RNA amplification of an E6/E7mRNA HPV assay was used in three studies (18, 19, 23).

### Meta-analysis

The pooled prevalence of HPV in abnormal cervical cytology.

The absence of publication bias was assessed with Egger’s regression test analysis (p = 0.125), which showed no publication bias. **Table 2** shows the Egger’s regression results.

**Table 2:** Egger’s regression results.

Std.-Eff.	Coef.	Std. Err	t	p>t	95% confidence interval	
Slope	5.022488	9.950689	0.50	0.622	-16.31962	26.36459
Bias	30.05708	18.41711	1.63	0.125	-9.443692	69.55784

**Figure 2:**
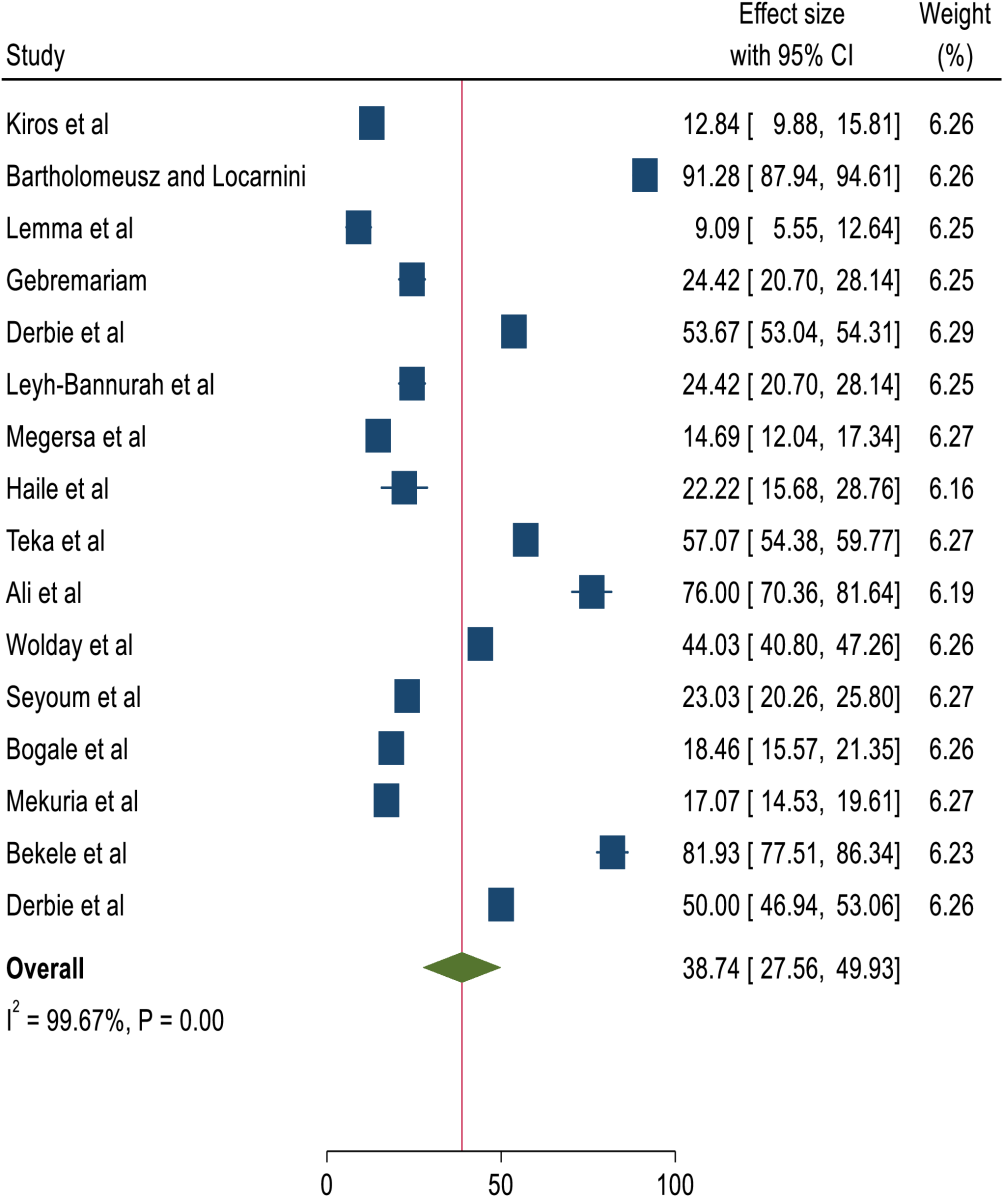
Forest plot of the pooled prevalence of HPV variant with abnormal cytology in Ethopia.

The overall pooled prevalence of HPV genotypes among women screened and identified with precancerous cervical lesions, either LSIL or HSIL, was 38.75%, with a 95% CI of 25.69-51.8 (4, 14, 18–31). **Figure 2** shows a forest plot of the pooled prevalence of HPV variants in Ethiopia.

### Subgroup analysis

Subgroup analysis was performed based on the study’s geographical region, HPV genotyping testing technique, and characteristics of the women. Based on the pooled effect of two or more studies, the three most common genotyping testing techniques identified were acetowhite changes by visual inspection with acetic acid (VIA) and HPV DNA testing for specimens in women with retrovirus infection (RVI) at 18%, all women screened for HPV at 12%, and women with RVI at 10% (4, 18, 19, 21, 23, 25, 26, 28, 29, 31). The least prevalent HPV type, at 6%, was found in two studies (24, 30). Among the genotyping test techniques used, the DNA test with amplification was the most commonly used test modality (14, 21, 22, 25–31). Central Ethiopia was the highest contributor to the HPV genotyping evidence, accounting for 66%, and the lowest were Oromia and southern Ethiopia, which accounted for 5% of the studies (4, 18–20). **Table 3** shows a subgroup analysis of the characteristics of the women, HPV testing technique, and region of Ethiopia.

**Table 3:** The subgroup analysis of the characteristics of the women, HPV testing technique, and region of Ethiopia.

Variable	Characteristics	Pooled prevalence(95% CI)	I^2^(P value)
**Characteristics of the women**	With abnormal cervical cytology	6%(3-13)	–
	Women screened for HPV	12%(10-15)	98.86(0.00)
	With abnormal cervical cytology and RVI	10%(7-14)	–
	With abnormal cervical cytology, but no RVI	6%(2-12)	93.74(0.00)
	With abnormal cervical cytology and acetowhite changes	18%(13-26)	82.44(0.00)
	With abnormal cytology and other STI complaints	7%(3-12)	95.55(0.00)
**Genotyping testing techniques**	Direct genome detection	5%(3-7)	68.90(0.00)
	DNA with amplification	14%(11-16)	98.97(0.00)
	RNA amplification of E6/E7	6%(4-8)	35.29(0.02)
**By region of Ethiopia**	Northwest	6%(2-10)	93.99(0.00)
	Armauer Hansen Res Center	12%(4-24)	98.08(0.00)
	Oromia	5%(3-7)	43.57(0.02)
	Tigray	9%(5-14)	71.14(0.00)
	Amhara	7%(3-13)	99.84(0.00)
	Southern Ethiopia	5%(3-7)	71.87(0.00)
	Central Ethiopia	66%(51-79)	93.66(0.00)
	South-central Ethiopia	22%(11-36)	96.64(0.00)
	Gynecology referral clinics	7%(4-10)	84.93(0.00)
	Eastern Ethiopia	7%(4-9)	79.93(0.00)
	Central Ethiopia	16%(12-20)	81.20(0.00)
	Southwest Ethiopia	8%(1-20)	96.41(0.00)

Among the 16 studies, there were 25 HPV variants identified by genotyping techniques in this review. The most prevalent HPV type was HPV-16 and HPV-18 coexisting (14) at 54%. The second most prevalent type was HPV-16 alone, accounting for 29% of the total number of studies. The third and fourth most common HPV types were HPV-51 and HPV-52, accounting for 16% and 13% (4, 14, 18, 19, 21–23, 25, 28–31) respectively. The fifth most prevalent HPV type were HPV-31 and HPV-33, each contributing approximately 12% of all HPV variant burdens in Ethiopia in previous studies (4, 14, 18, 19, 21, 22, 25–31). **Table 4** shows the prevalence of HPV DNA test results by subgroup analysis of HPV DNA genotypes in Ethiopia.

**Table 4:** The prevalence of HPV DNA test results by subgroup analysis of HPV DNA genotypes in Ethiopia.

Variable	HPV type	Pooled prevalence (95% CI)	I^2^(P value)
HPV DNA genotype	16	29% (19-41)	97.69(0.00)
	18	5%(3-7)	78.72(0.00)
	31	12%(7-18)	94.75(0.00)
	33	12%(3-26)	96.12(0.00)
	35	11%(6-18)	95.20(0.00)
	39	11%(4-22)	94.69(0.00)
	45	10%(5-16)	94.16(0.00)
	52	13%(7-19)	94.98(0.00)
	58	10%(4-17)	95.90(0.00)
	66	10%(1-26)	96.67(0.00)
	68	11%(4-19)	95.86(0.00)
	39 and 68	3%(1-7)	–
	56 and 74	1%(0-4)	–
	6	5%(1-11)	86.23(0.00)
	51	16%(6-31)	94.14(0.00)
	56	10%(5-17)	94.44(0.00)
	59	7%(7-14)	94.61(0.00)
	53	2%(0-5)	–
	35 and 39	6%(3-13)	–
	45 and 68	5%(2-11)	–
	16 and 18	54%(52-55)	–
	11	7%(0-24)	–
	42	2%(1-2)	–
	70	2%(0-6)	–
	68 and 73	2%(1-8)	–

### Sensitivity analysis

Two studies (27), and (29), had an impact on the overall estimation of the meta-analysis results. **Supplementary File 3** shows the sensitivity analysis.

## Discussion

According to our review and meta-analysis, the prevalence any HPV genotype being detected among women who had a precancerous cervical lesion in health facilities was 38.75% (25.69-51.81). A recent meta-analysis showed that the proportion of patients infected with HR-HPV was 42.2% in Eastern Africa (32). Similarly, the prevalence of HPV in women with precancerous cervical lesions and cervical cancer was between 13.7% and 93%. A globally based review showed that the prevalence of HPV genotype was between 11% and 12% (with higher rates, 24%, in sub-Saharan Africa) in women without cervical abnormalities (15, 31, 33).

The detection of HPV increases in women with abnormal cervical cytology in proportion to the severity of the lesions, which supports our findings (33). Based on a global review, HPV was detected 90% of the time in abnormal cervical cytology, which is relatively higher than that reported in this review and meta-analysis (33). This might be because developed nations use more sophisticated DNA testing techniques than Ethiopia. In addition, a review showed that the prevalence of HPV was 84.8% among Asian patients with atypical squamous cell lesions (34).

The predominant genotypes identified in this review were HPV-16 and HPV-18, accounting for 9.52% and 8.33%, respectively; HPV-31 and HPV-48, each accounting for 7.74%; HPV-52 and HPV-56, each accounting for 7.14%; and HPV-35, HPV-58, and HPV-59 with an average of 6.55% of all HPV variant burdens in Ethiopia. These findings are essential for predicting how HPV vaccination and HPV-based screening will impact cervical cancer prevention in Ethiopia. This infers that further HPV vaccine studies in Ethiopia should mainly target the most predominant genotypes as the current vaccine type only targets HPV-6, HPV-11, HPV-16, and HPV-18.

Based on similar review reports in different parts of the globe, the genotype distributions of HPVs in different countries from different kinds of cervical lesions were compared with this review. HPV-16 is the most common genotype consistently reported globally as an important cause of cervical abnormalities (35–37). The pooled prevalence of HPV-16 sub genotypes in this review and meta-analysis was found to be 29% which was relatively comparable with the overall incidence in Africa (35). The pooled prevalence of subgroup HPV genotypes 31 and 33 were 12% each, which was comparable to (35), which found 8.2% and 10.3%, respectively. However, HPV types 51 and 52 were not reported as prevalent genotypes, but in this study, they were the most prevalent cases, accounting for 16% (6–31) and 13% (7–19), respectively. HPV genotype 18 was among the lowest at 5% and HPV genotype 35 was among the dominant at 11% in this review.

This variation in HPV genotype distribution across the studies is likely attributable to differences in the population, severity of cervical lesions, age at screening initiation, frequency, coverage, and follow-up rates of women with cervical abnormalities (38). In addition, the difference might also be associated with ethnic differences, geographical location, and the sexual behavior of their male partners (39).

Globally, HPV type-specific prevalence varies. A study in Asia (40) indicated that HPV-16 was most prevalent at 23.9%, which was comparable with the findings of this review. In contrast, the prevalence of HPV type 52 was lower than that in this review. However, studies in North America (37) found the prevalence of HPV types 16, 31, and 51 to be 26%, 11.5%, and 10.6%, respectively. These findings are comparable to those of this review and meta-analysis. Similarly, in a study in Israel (41), the most prevalent HPV type was HPV-16 (46.5%), which is higher than that of this review and meta-analysis. However, the prevalence of HPV type 31 was comparable at 7%. These differences among the studies and this review might be due to variations in population, DNA testing technique, and sampling technique.

According to the results of this review and meta-analysis, the pooled prevalence of HPV 16/18 in the combined subgroup was 54%. Similarly, other reviews showed that 45.1% of HPV16/18 combined were from high-grade cervical lesions, while 67.7% were from abnormal cervical cytology among African women and 605 Israeli women (15, 31, 35, 41). Among HPV-positive patients, the co-existing prevalence of HPV 16/18 was reported differently in different countries, as it was 87.5% in Central and Eastern Europe (42) and 80% in India among those with high-grade cervical lesions (43).

HPV-16 was the most prevalent type in this review at 29% (19%-41%), and HPV-18 was not among the five most prevalent types at 5% (3%-7%). These findings were comparable to those of studies in Italy on HPV-18 (7%). However, HPV-16 (64%) was much more prevalent than this review finding (44). A review by Guan et al. revealed that HPV-16 positivity increased steeply from normal to high-grade cervical lesions (44). Accordingly, vaccine mixes and HPV-based screening tests should always include this genotype, although some low-grade cervical lesions associated with certain other HPVs may preferentially progress to cervical cancer (3, 15). Our review envisages a future impact of the broadly identified subgroup pooling of the genotypes (HPV-16 and HPV-18 coexisting, HPV-16, HPV-51, HPV-52, HPV-31, and HPV-33) on vaccination and HPV-based screening in Ethiopia.

The Ethiopian Ministry of Health started vaccinating schoolchildren aged 14 years using Gardasil-4™ (HPV-6, HPV-11, HPV-16, HPV-18) in 2018. However, in this review, in addition to the genotypes covered by the current vaccine, there were other genotypes found to be prevalent in Ethiopia, such as HPV-51, HPV-52, HPV-31, and HPV-32. Hence, HPV-based screening based on the detection of HPV16/18 oncoproteins and, most recently, the use of the HPV DNA test has been employed. This finding suggests that vaccinating girls using Gardasil-4TM and screening women for cervical lesions using HPV16/18 oncoproteins significantly reduces the number of girls who might be protected.

The vaccine for girls would be more effective if the most prevalent genotype distribution was included. This is because women might be missed by screening programs for the most dominant HPV genotypes circulating in the country. For this reason, most developed countries are currently using other vaccine types of the monovalent Gardasil^®^9 (6, 11, 16, 18, 31, 33, 45, 52, and 58) vaccine that targets close to 90% of all HR-HPVs (45), which is essentially an ideal type of vaccine for Ethiopians based on our review findings. However, this vaccine type might not cover all the top five highly distributed HPV genotypes, except for HPV-52. Even though there is a financial limitation, the nonavalent vaccine type would be the one that includes all the most prevalent HPV genotypes, including HPV-51, for the Ethiopian setting.

### Strengths and limitations

This systematic review and meta-analysis was the first to analyze results from women with abnormal cervical cytology, and important input will be obtained to revise the current vaccination and HPV-based screening program in Ethiopia. The review included studies from different health facilities and geographical areas, with a wide range of study participants and different DNA tests and techniques, which enabled us to obtain a better picture of the HPV genotype burden in Ethiopia. This review and meta-analysis result should be interpreted in light of several limitations. Because of the absence of articles on women’s abnormal cervical cytology test results in some parts of the country, our findings could compromise the overall picture of the HPV genotype distribution in Ethiopia.

## Conclusion

In this review and meta-analysis, HPV genotypes were predominantly identified from different kinds of cervical samples via abnormal cervical cytology. There are currently two types of HPV vaccines licensed in many countries, and these vaccines have been proven to prevent more than 95% of HPV infections caused by HPV types 16 and 18, which cause 70% of cancer cases, but the HPV genome distribution is not uniform across the country. The pooled prevalence of HPV genotypes in Ethiopia was greater than that in the other countries. HPV-16 and HPV-18 coexist, and HPV-16, HPV-51, HPV-52, HPV-31, and HPV-33 are the most prevalent HPV genotypes which require special attention when designing vaccination and HPV-based cervical cancer screening programs. Additional data on similar DNA test techniques among women with cervical cancer are needed. It is important to place emphasis on the nationwide HPV distribution in the prevention and control strategies.

## Data Availability

The data will be available upon reasonable request to corresponding author. Requests to access these datasets should be directed to SK, solomondemis@gmail.com.

## References

[1] WHO . Global Cancer Observatory Ethiopia Fact Sheet Vol. 133. Geneva, Switzerland: Int agency Res cancer (2021) p. 2020–1.

[2] Ruland R Prugger C Schiffer R Regidor M Lellé RJ . Prevalence of human papilloma virus infection in women in rural Ethiopia. Eur J Epidemiol. (2006) 21:727–9. doi: 10.1007/s10654-006-9055-4 17072541

[3] Menon S Wusiman A Boily MC Kariisa M Mabeya H Luchters S . Epidemiology of HPV genotypes among HIV positive women in Kenya: A systematic review and meta-analysis. PloS One. (2016) 11. doi: 10.1371/journal.pone.0163965 PMC5072621 27764092

[4] Leyh-Bannurah SR Prugger C De Koning MNC Goette H Lellé RJ . Cervical human papillomavirus prevalence and genotype distribution among hybrid capture 2 positive women 15 to 64 years of age in the Gurage zone, rural Ethiopia. Infect Agent Cancer. (2014) 9:1–9. doi: 10.1186/1750-9378-9-33 25320636 PMC4197284

[5] World Health Organization . Cervical cancer profile Vol. 2020). Geneva, Switzerland: World Heal Organ (2021). p. 2021.

[6] Federal Minstry of Health E . NATIONAL CERVICAL CANCER PREVENTION TRAINING PACKAGE PARTICIPANT MANUAL. Addis Ababa: Federal Ministry of Health (2022).

[7] FMOH . Guideline for Cervical Cancer Prevention and Control in Ethiopia Vol. 35. Addis Ababa: Federal Ministry of Health (2015).

[8] Joste NE Rushing L Granados R Zitz JC Genest DR Crum CP . Bethesda classification of cervicovaginal smears: Reproducibility and viral correlates. Hum Pathol. (1996) 27:581–5. Available online at: https://www.sciencedirect.com/science/article/pii/S0046817796901653. 10.1016/s0046-8177(96)90165-3 8666368

[9] Genest DR Stein L Cibas E Sheets E Zitz JC Crum CP . A binary (Bethesda) system for classifying cervical cancer precursors: Criteria, reproducibility, and viral correlates. Hum Pathol. (1993) 24:730–6. Available online at: https://www.sciencedirect.com/science/article/pii/0046817793900096. 10.1016/0046-8177(93)90009-6 8391511

[10] Berek JS . Simplification of the new Bethesda 2001 classification system. Am J Obstet Gynecol. (2003) 188:S2–5. Available online at: https://www.sciencedirect.com/science/article/pii/S0002937802715408. 10.1067/mob.2003.220 12634623

[11] Skinner SR Wheeler CM Romanowski B Castellsagué X Lazcano-Ponce E Del Rosario-Raymundo MR . Progression of HPV infection to detectable cervical lesions or clearance in adult women: Analysis of the control arm of the VIVIANE study. Int J cancer. (2016) 138:2428–38. doi: 10.1002/ijc.29971 PMC4787275 26685704

[12] Welby S Rosillon D Feng Y Borys D . Progression from human papillomavirus (HPV) infection to cervical lesion or clearance in women (18-25 years): Natural history study in the control arm subjects of AS04-HPV-16/18 vaccine efficacy study in China between 2008 and 2016. Expert Rev Vaccines. (2022) 21:407–13. doi: 10.1080/14760584.2022.2021077 34939897

[13] Jahic M Kameric L Hadzimehmedovic A . Progression low squamous intraepithelial lesion and human papillomavirus infections. Mater Sociomed. (2020) 32:127–30. doi: 10.5455/msm. PMC7428887 32843861

[14] Derbie A Maier M Amare B Misgan E Nibret E Liebert UG . High − risk human papillomavirus genotype distribution among women with gynecology complaints in northwest Ethiopia. Infect Agent Cancer. (2023), 1–10. doi: 10.1186/s13027-023-00481-3 36703179 PMC9881258

[15] Derbie A Mekonnen D Yismaw G Biadglegne F Van Ostade X Abebe T . Human papillomavirus in Ethiopia. VirusDisease. (2019) 30:171–9. doi: 10.1007/s13337-019-00527-4 PMC6531598 31179353

[16] Stroup DF Berlin JA Morton SC Olkin I Williamson GD Rennie D . Meta-analysis of observational studies in epidemiology: a proposal for reporting. Meta-analysis Of Observational Studies in Epidemiology (MOOSE) group. JAMA. (2000) 283:2008–12. doi: 10.1001/jama.283.15.2008 10789670

[17] Munn Z Stone JC Aromataris E Klugar M Sears K Leonardi-Bee J . Assessing the risk of bias of quantitative analytical studies: introducing the vision for critical appraisal within JBI systematic reviews. JBI Evid Synth. (2023) 21. Available online at: https://journals.lww.com/jbisrir/fulltext/2023/03000/assessing_the_risk_of_bias_of_quantitative.2.aspx. 10.11124/JBIES-22-00224 36476419

[18] Haile EL Cindy S Ina B Belay G Jean-Pierre VG Sharon R . HPV testing on vaginal/cervical nurse-assisted self-samples versus clinician-taken specimens and the HPV prevalence, in Adama Town, Ethiopia. Med (Baltimore). (2019) 98:e16970. doi: 10.1097/MD.0000000000016970 PMC6736428 31464941

[19] Lemma E . Evaluation of cervical cancer screening uptake, HPV genotyping and self-sampling collection techniques. Antwerp, Belgium: University of Antwerp (2022). pp. 1–155.

[20] Megersa T Dango S Kumsa K Lemma K Lencha B . Prevalence of high − risk human papillomavirus infections and associated factors among women living with HIV in Shashemene town public health facilities, Southern Ethiopia. BMC Womens Health. (2023), 1–10. doi: 10.1186/s12905-023-02279-2 36959649 PMC10036163

[21] Ali KE Mohammed IA Difabachew MN Demeke DS Haile T Ten Hove RJ . Burden and genotype distribution of high-risk Human Papillomavirus infection and cervical cytology abnormalities at selected obstetrics and gynecology clinics of Addis Ababa, Ethiopia. BMC Cancer. (2019) 19:1–9. doi: 10.1186/s12885-019-5953-1 31382907 PMC6683490

[22] Bogale AL Teklehaymanot T Kassie GM Medhin G Ali JH Belay NB . Performance of visual inspection with acetic acid for cervical cancer screening as compared to human papillomavirus deoxyribonucleic acid testing among women with HIV in ethiopia: a comparative cross-sectional study. Cancer Control. (2022) 29:1–7. doi: 10.1177/10732748221114980 PMC9284200 35829643

[23] Mekuria SF Jerkeman M Forslund O Fikru S Borgfeldt C . Detection of HPV mRNA in self-collected vaginal samples among urban Ethiopian women. Anticancer Res. (2020) 40:1513–7. doi: 10.21873/anticanres.14096 32132051

[24] Kiros M Belay DM Getu S Hailemichael W Esmael A Andualem H . Prevalence and determinants of pre-cancerous cervical lesion and human papillomavirus among hiv-infected and hiv-uninfected women in north-west Ethiopia: A comparative retrospective cross-sectional study. HIV/AIDS - Res Palliat Care. (2021) 13:719–25. doi: 10.2147/HIV.S310905 PMC8254534 34234573

[25] Gebremariam T . Human papillomavirus related cervical cancer and anticipated vaccination challenges in Ethiopia. Int J Heal Sci. (2016) 10:137–43. doi: 10.12816/0031220 PMC4791164 27004064

[26] Teka B Gizaw M Ruddies F Addissie A Chanyalew Z Skof AS . Population-based human papillomavirus infection and genotype distribution among women in rural areas of South Central Ethiopia. Int J Cancer. (2021) 148:723–30. doi: 10.1002/ijc.33278 32875552

[27] Bekele A Baay M Mekonnen Z Suleman S Chatterjee S . Human papillomavirus type distribution among women with cervical pathology - A study over 4 years at Jimma Hospital, southwest Ethiopia. Trop Med Int Heal. (2010) 15:890–3. doi: 10.1111/tmi.2010.15.issue-8 20545913

[28] Seyoum A Seyoum B Gure T Alemu A Belachew A Abeje D . Genotype heterogeneity of high-risk human papillomavirus infection in Ethiopia. Front Microbiol. (2023) 14:1–10. doi: 10.3389/fmicb.2023.1074826 PMC9951590 36846744

[29] Bartholomeusz A Locarnini S . Associated with antiviral therapy. Antivir Ther. (2006) 55:52–5.

[30] Wolday D Derese M Gebressellassie S Tsegaye B Ergete W Gebrehiwot Y . HPV genotype distribution among women with normal and abnormal cervical cytology presenting in a tertiary gynecology referral Clinic in Ethiopia. Infect Agent Cancer. (2018) 13:4–11. doi: 10.1186/s13027-018-0201-x 30127841 PMC6092870

[31] Derbie A Mekonnen D Nibret E Maier M Woldeamanuel Y Abebe T . Human papillomavirus genotype distribution in Ethiopia: an updated systematic review. Virol J. (2022) 19:4–11. doi: 10.1186/s12985-022-01741-1 35033141 PMC8760777

[32] Kiyingi J Nabunya P Bahar OS Mayo-LJ Id YT Nabayinda J . Prevalence and predictors of HIV and sexually transmitted infections among vulnerable women engaged in sex work : Findings from the Kyaterekera Project in Southern Uganda. PloS One. (2022), 1–14. doi: 10.1371/journal.pone.0273238 PMC9522279 36174054

[33] Formana D de Martel C Lacey CJ Soerjomatarama I Lortet-Tieulent J Bruni L . Global burden of human papillomavirus and related diseases. Vaccine. (2012) 30:F12–23. doi: 10.1016/j.vaccine.2012.07.055 23199955

[34] Bruni L Albero G Serrano B Mena M Collado JJ Gómez D . Human Papillomavirus and Related Diseases in Ethiopia. Lyon, France (IARC) and Barcelona, Spain (ICO): Catalan Institute of Oncology (ICO) and the International Agency for Research on Cancer (IARC) (2023).

[35] Ogembo RK Gona PN Seymour AJ Park HSM Bain PA Maranda L . Prevalence of human papillomavirus genotypes among African women with normal cervical cytology and neoplasia: A systematic review and meta-analysis. PloS One. (2015) 10:1–22. doi: 10.1371/journal.pone.0122488 PMC4396854 25875167

[36] Shavit O Roura E Barchana M Diaz M Bornstein J . Burden of human papillomavirus infection and related diseases in Israel. Vaccine. (2013) 31:I32–41. Available online at: https://www.sciencedirect.com/science/article/pii/S0264410X13009213. 10.1016/j.vaccine.2013.05.108 24229717

[37] Clifford GM Rana RK Franceschi S Smith JS Gough G Pimenta JM . Human papillomavirus genotype distribution in low-grade cervical lesions: Comparison by geographic region and with cervical cancer. Cancer Epidemiol Biomarkers Prev. (2005) 14:1157–64. doi: 10.1158/1055-9965.EPI-04-0812 15894666

[38] Derbie A Amare B Misgan E Nibret E Maier M Woldeamanuel Y . Histopathological profile of cervical punch biopsies and risk factors associated with high-grade cervical precancerous lesions and cancer in northwest Ethiopia. PloS One. (2022) 17:1–15. doi: 10.1371/journal.pone.0274466 PMC9467373 36094938

[39] Davarmanesh M Dezfulian M Gharavi MJ Younesi S Saadati P Amin MMT . Human papilloma virus (HPV) genotypes concordance between Iranian couples referrals. Infect Agent Cancer. (2019) 14:1–8. doi: 10.1186/s13027-019-0241-x 31516545 PMC6734344

[40] Peng R-R Li H-M Chang H Li J-H Wang AL Chen X-S . Prevalence and genotype distribution of cervical human papillomavirus infection among female sex workers in Asia: a systematic literature review and meta-analysis. Sex Health. (2012) 9:113–9. doi: 10.1071/SH11066 PMC6322668 22498154

[41] Shavit O Roura E Barchana M Diaz M Bornstein J . Burden of human papillomavirus infection and related diseases in Israel. Vaccine. (2013) 31:132–41. doi: 10.1016/j.vaccine.2013.05.108 24229717

[42] Poljak M Seme K Maver PJ Kocjan BJ Cuschieri KS Rogovskaya SI . Human papillomavirus prevalence and type-distribution, cervical cancer screening practices and current status of vaccination implementation in central and eastern Europe. Vaccine. (2013) 31. doi: 10.1016/j.vaccine.2013.03.029 24332298

[43] Sankaranarayanan R Bhatla N Gravitt PE Basu P Esmy PO Ashrafunnessa KS . Human papillomavirus infection and cervical cancer prevention in India, Bangladesh, Sri Lanka and Nepal. Vaccine. (2008) 26. doi: 10.1016/j.vaccine.2008.05.005 18945413

[44] Di Felice E Caroli S Paterlini L Campari C Prandi S Rossi PG . Cervical cancer epidemiology in foreign women in Northern Italy: Role of human papillomavirus prevalence in country of origin. Eur J Cancer Prev. (2015) 24:223–30. doi: 10.1097/CEJ.0000000000000137 25714783

[45] Zhai L Tumban E . Gardasil-9: A global survey of projected efficacy. Antiviral Res. (2016) 130:101–9. doi: 10.1016/j.antiviral.2016.03.016 27040313

