# Supplementary material for: Prevalence and distribution of human papillomavirus genotypes in women with abnormal cervical cytology in Ethiopia: a systematic review and meta-analysis
Source: Front Oncol. 2024 Oct 15;14:1384994. doi: 10.3389/fonc.2024.1384994 (PMC11518683; doi:10.3389/fonc.2024.1384994)
Supplement: Supplementary file 1 [file DataSheet1.docx]

**Supplementary file 1:** MOOSE checklist for meta-analysis of observational studies

| **Item No** | **Recommendation** | | | |
| --- | --- | --- | --- | --- |
| Reporting of background should include | | | |  |
| 1 | Problem definition | | | |
| 2 | Hypothesis statement | | | |
| 3 | Description of study outcome(s) | | | |
| 4 | Type of exposure or intervention used | | | |
| 5 | Type of study designs used | | | |
| 6 | Study population | | | |
| Reporting of search strategy should include | | | |  |
| 7 | Qualifications of searchers (eg, librarians and investigators) | | | |
| 6 | Search strategy, including time period included in the synthesis and key words | | | |
| 6 | Effort to include all available studies, including contact with authors | | | |
| 6 | Databases and registries searched | | | |
| 6 | Search software used, name and version, including special features used (eg,  explosion) | | | |
| 6 | Use of hand searching (eg, reference lists of obtained articles) | | | |
| - | List of citations located and those excluded, including justification | | | |
| 6 | Method of addressing articles published in languages other than English | | | |
| - | Method of handling abstracts and unpublished studies | | | |
| - | Description of any contact with authors | | | |
| - | | | |  |
| 17 | Description of relevance or appropriateness of studies assembled for assessing the  hypothesis to be tested | | | |
| 18 | Rationale for the selection and coding of data (eg, sound clinical principles or  convenience) | | | |
| 19 | Documentation of how data were classified and coded (eg, multiple raters, blinding  and interrater reliability) | | | |
| 20 | Assessment of confounding (eg, comparability of cases and controls in studies where  appropriate) | | | |
| 21 | Assessment of study quality, including blinding of quality assessors, stratification or  regression on possible predictors of study results | | | |
| 22 | Assessment of heterogeneity | | | |
| 23 | Description of statistical methods (eg, complete description of fixed or random effects models, justification of whether the chosen models account for predictors of study results, dose-response models, or cumulative meta-analysis) in sufficient detail  to be replicated | | | |
| 24 | Provision of appropriate tables and graphics | | | |
| Reporting of results should include | | | |  |
| 25 | Graphic summarizing individual study estimates and overall estimate | | | |
| 26 | Table giving descriptive information for each study included | | | |
| 27 | Results of sensitivity testing (eg, subgroup analysis) | | | |
| 28 | Indication of statistical uncertainty of findings | | | |
| Item No | Recommendation | | | |
| Reporting of discussion should include | | |  |  |
| 29 | | Quantitative assessment of bias (eg, publication bias) |  |  |
| 30 | | Justification for exclusion (eg, exclusion of non-English language citations) |  |  |
| 31 | | Assessment of quality of included studies |  |  |
| Reporting of conclusions should include | | |  |  |
| 32 | | Consideration of alternative explanations for observed results |  |  |
| 33 | | Generalization of the conclusions (ie, appropriate for the data presented and within the  domain of the literature review) |  |  |
| 34 | | Guidelines for future research |  |  |
| 35 | | Disclosure of funding source |  |  |
