# Supplementary material for: Prevalence and distribution of human papillomavirus genotypes in women with abnormal cervical cytology in Ethiopia: a systematic review and meta-analysis
Source: Front Oncol. 2024 Oct 15;14:1384994. doi: 10.3389/fonc.2024.1384994 (PMC11518683; doi:10.3389/fonc.2024.1384994)
Supplement: Supplementary file 2 [file DataSheet2.docx]

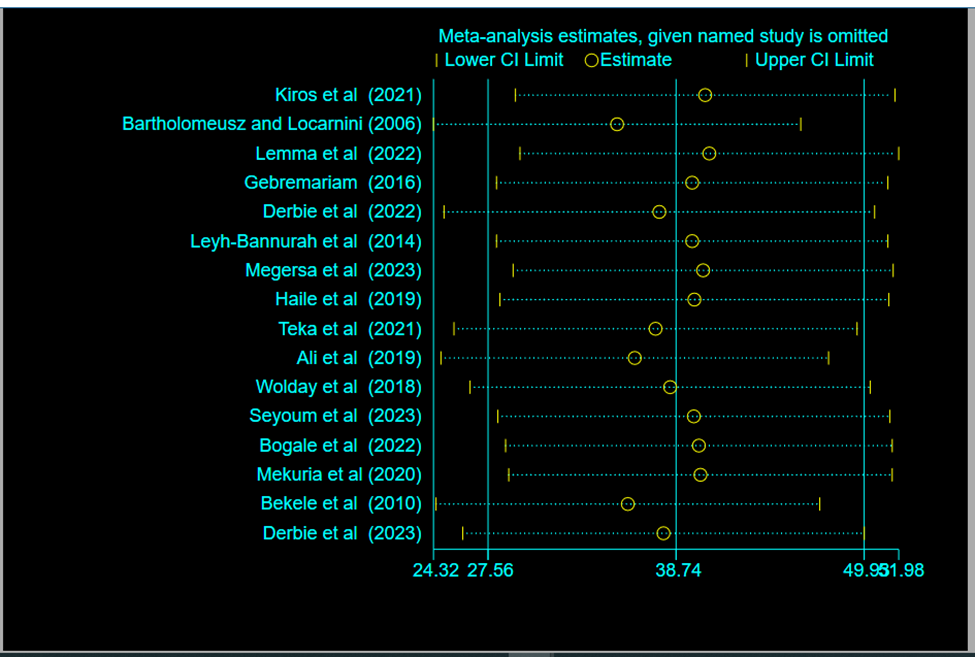


**Supplementary file 2:** The overall pooled prevalence sensitivity analysis of Prevalence of HPV genotyping among abnormal cytology when the studies omitted step by step
