# Supplementary material for: Prevalence and distribution of human papillomavirus genotypes in women with abnormal cervical cytology in Ethiopia: a systematic review and meta-analysis
Source: Front Oncol. 2024 Oct 15;14:1384994. doi: 10.3389/fonc.2024.1384994 (PMC11518683; doi:10.3389/fonc.2024.1384994)
Supplement: Supplementary file 3 [file DataSheet3.docx]

Supplementary file 3: Quality Assessment of the Included Studies Using the Joanna Briggs Institute (JBI) Quality Appraisal Criteria

| Author | Criteria | | | | | | | | | Score | Quality |
| --- | --- | --- | --- | --- | --- | --- | --- | --- | --- | --- | --- |
|  | Was The Sample Frame Appropriate? | Was Sampling Appropriate? | Was The Sample Size Adequate? | Were The Study Subjects And The Setting Described In Detail? | Was The Data Analysis Conducted With Sufficient Coverage Of The Identified Sample? | Were Valid Methods Used For The Identification Of The Condition? | Was The Condition Measured In A Standard, Reliable Way For All Participants? | Was There Appropriate Statistical Analysis? | Was The Response Rate Adequate, And If Not, Was The Low Response Rate Managed Appropriately? |  |  |
| Bagga *et Al.*, 2023 | yes | yes | yes | yes | yes | yes | no | yes | no | 6 | Low risk |
| Chien *Et Al.*, 2018 | yes | yes | no | yes | no | yes | yes | yes | no | 6 | Low risk |
| Gidi *Et Al*., 2020 | yes | yes | yes | yes | yes | yes | yes | yes | yes | 9 | Low risk |
| Figueras-Aloy *Et Al.*, 2020 | yes | yes | yes | yes | yes | yes | no | yes | no | 6 | Low risk |
| Hemmati And Ghassemzadeh, 2023 | yes | yes | yes | yes | not clear | yes | yes | yes | yes | 8 | Low risk |
| Hsu *et al.*, 2018 | yes | yes | yes | yes | yes | yes | no | yes | no | 6 | Low risk |
| Iacobelli *et al.*, 2015 | yes | yes | yes | yes | yes | yes | yes | yes | yes | 9 | Low risk |
| Liao *et al.*, 2019 | yes | yes | yes | yes | yes | yes | no | yes | no | 6 | Low risk |
| Lima, 2016 | Yes | Yes | Yes | Yes | Yes | Yes | Yes | Yes | Yes | 9 | Low risk |
| Looney And Adamkin, 2003 | yes | yes | yes | no | yes | yes | no | yes | yes | 7 | Low risk |
| Lunde D And Rnc DNP, 2014 | yes | yes | yes | not clear | yes | yes | yes | yes | yes | 8 | Low risk |
| Makker, 2021 | yes | yes | yes | yes | yes | yes | yes | yes | yes | 9 | Low risk |
| Perrin *et al.*, 2023 | yes | yes | yes | yes | yes | yes | no | yes | no | 6 | Low risk |
| Raturi *Et Al.*, 2017 | yes | yes | yes | yes | yes | yes | yes | yes | yes | 9 | Low risk |
| Rover *Et Al.*, 2016 | yes | yes | yes | yes | yes | yes | yes | yes | yes | 9 | Low risk |
| Shen *et al.*, 2022 | yes | yes | yes | yes | yes | yes | no | yes | no | 6 | Low risk |
| Umberto *et al.*, 2021 | yes | yes | yes | yes | no | yes | no | yes | yes | 7 | Low risk |
